# An Inkjet Printed Flexible Electrocorticography (ECoG) Microelectrode Array on a Thin Parylene-C Film

**DOI:** 10.3390/s22031277

**Published:** 2022-02-08

**Authors:** Yoontae Kim, Stella Alimperti, Paul Choi, Moses Noh

**Affiliations:** 1 American Dental Association Science & Research Institute, Gaithersburg, MD 20899, USA; kimyo@ada.org (Y.K.); alimpertis@ada.org (S.A.); 2 Department of Electrical and Computer Engineering, University of Texas Rio Grande Valley, Edinburg, TX 78539, USA; Paul.choi@utrgv.edu; 3 Department of Mechanical Engineering and Mechanics, Drexel University, Philadelphia, PA 19104, USA

**Keywords:** inkjet printing, ECoG sensor, microelectrode array, Parylene-C, flexible substrate, neural probe

## Abstract

Electrocorticography (ECoG) is a conventional, invasive technique for recording brain signals from the cortical surface using an array of electrodes. In this study, we developed a highly flexible 22-channel ECoG microelectrode array on a thin Parylene film using novel fabrication techniques. Narrow (<40 µm) and thin (<500 nm) microelectrode patterns were first printed on PDMS, then the patterns were transferred onto Parylene films via vapor deposition and peeling. A custom-designed, 3D-printed connector was built and assembled with the Parylene-based flexible ECoG microelectrode array without soldering. The impedance of the assembled ECoG electrode array was measured in vitro by electrochemical impedance spectroscopy, and the result was consistent. In addition, we conducted in vivo studies by implanting the flexible ECoG sensor in a rat and successfully recording brain signals.

## 1. Introduction

Various neurophysiological techniques have been proposed for monitoring the brain’s electrical signals [1,2,3,4]. Electrocorticography (ECoG) is an invasive but non-penetrating technique that uses an array of electrodes to monitor responses evoked from the brain’s surface. ECoG offers several practical advantages over penetrative techniques, such as a high spatial resolution [3,5,6] and signal quality [7] due to direct contact with tissues. In addition, the ECoG recording system reduces undesirable reactions, such as mechanical trauma [8], foreign body reactions [9], and any damages caused by the mechanical mismatch [10] during in vivo recording. 

A variety of polymeric materials, such as Polydimethylsiloxane (PDMS), Polyimide (PI), and Parylene, have been considered for the creation of highly flexible ECoG electrode arrays [11,12,13,14,15]. Compared with ECoG electrode arrays made of rigid materials, polymeric materials have a low elastic modulus that is advantageous for in vivo signal recording. A flexible ECoG electrode array can be placed on a wrinkled surface through a small opening in the skull. Therefore, the trauma of skull opening and damage to the brain surface by mechanical discrepancies between substrates and the feeble brain can be minimized [1,4,6,16].

PDMS has the lowest elastic modulus among the polymers mentioned above, and its elastic modulus is only two orders of magnitude higher than that of brain tissues [17,18]. PDMS is widely used in neurophysiological applications, but it is challenging to make thin-film devices [19,20,21]. Polyimide is an excellent thin-film material, and it is easy to control its thickness. However, the elastic modulus of Polyimide is higher than other polymer materials [16,22,23]. Parylene has been used to build flexible devices for biomedical applications [24,25,26,27,28,29,30]. The elastic modulus of Parylene is lower than that of Polyimide and its unique properties, such as optical transparency [3], biocompatibility [31], and flexibility, have made it attractive in neurophysiological applications.

Conventional microfabrication techniques such as photolithography, thin-film deposition, etching, and lift-off have been used by other researchers to make ECoG microelectrodes array on both inorganic (rigid) and organic (flexible) materials [2,26,27,28,29]. However, these methods are time-consuming, costly, and require cleanroom facilities and hazardous chemicals. Inkjet printing can be an alternative fabrication method that can create narrow and thin microelectrode patterns using various kinds of conductive inks. Thus, inkjet printing has great potential as a rapid, low-cost, and mask-free fabrication method for ECoG electrodes array on flexible materials [32,33,34,35,36]. We have previously reported a novel method of fabricating thin flexible Parylene-based microelectrode arrays using inkjet printing and silver nanoparticle-based ink for electrokinetic applications [37].

In this paper, we report on a thin Parylene-based flexible 22-channel ECoG microelectrode array made by inkjet printing technique. Narrow (<40 µm) and thin (<500 nm) microelectrode patterns were first printed on PDMS, then transferred onto a Parylene film via vapor deposition and release. To achieve a higher electrical conductivity, we deposited a thin copper film on the printed silver electrodes using electroplating. Thereafter, a thin layer of Parylene was deposited onto the electrode patterns, followed by ArF excimer laser machining for creating micro-sensing sites (80 × 80 µm) through the thin Parylene layer. Finally, a thin gold film was selectively deposited on the sensing sites using electroless plating for in vivo animal experiments. A custom-designed, 3D-printed connector was built and assembled with the Parylene-based flexible ECoG microelectrode array, without soldering. The impedance of the assembled ECoG electrode array was measured in vitro by electrochemical impedance spectroscopy, and the result was found to be consistent. In addition, we implanted the printed ECoG sensor in a rat and successfully recorded brain signals. We believe that the Parylene-based flexible microelectrode patterns created by the inkjet printing technique, as described here, can find a myriad of applications in the field of neuroscience and brain–machine interfaces.

## 2. Materials and Methods

### 2.1. ECoG Electrode Design and Inkjet Printing

**Figure 1:**
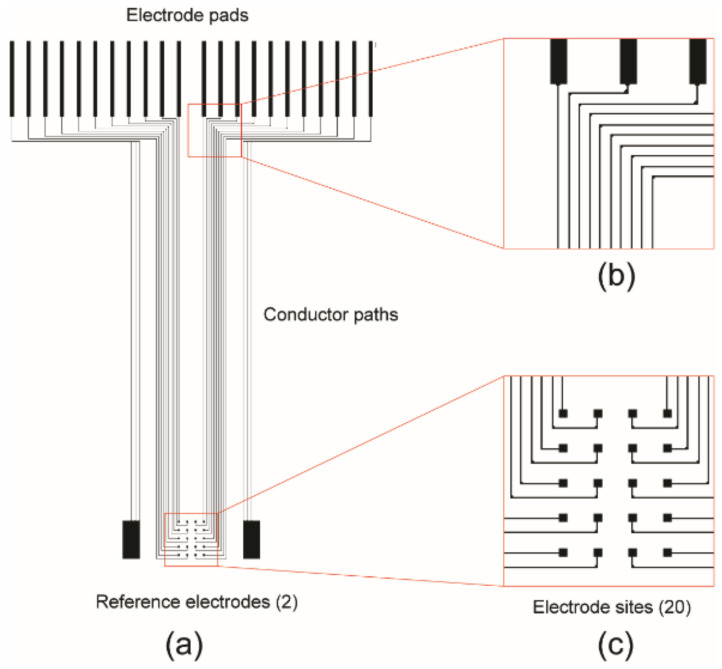
ECoG electrode design: (**a**) CAD drawing of inkjet printing pattern of the ECoG electrode array with 22 electrode pads (top), the conductor paths (middle), 20 electrode sites, and 2 references electrodes (bottom); (**b**) detailed view of the connection between conductor paths and electrode pads and (**c**) 4 × 5 sensing electrodes.

The ECoG electrode design for the current study is shown in Figure 1. There are 20 sensing electrodes and 2 reference electrodes connected to electrode pads (Figure 1a,b). The overall size of the electrode pattern is 31 mm × 22 mm. The 20 sensing electrodes are arranged in a 4 × 5 matrix with sensing areas of 100 µm × 100 µm, and the pitch is 500 µm (Figure 1c). Two reference electrodes are located at both sides of the matrix area. Each reference electrode has an area of 2.25 mm^2,^ and is several orders of magnitude larger than the sensing electrodes. The dimension of each electrode pad is 0.22 mm × 4.5 mm. The pitch between the pads is 1 mm.

We used a drop-on-demand (DOD) inkjet printer (DMP-2800, Fujifilm, Tokyo, Japan) with one picoliter drop-volume piezoelectric printer head (DMC-11601, Fujifilm, Tokyo, Japan) for the deposition of silver nanoparticle ink onto the PDMS substrates. We used a fixed jetting frequency of 2 kHz, and the nozzle and platen temperatures were fixed at 30 °C. The drop velocity was 8.0 m·s^−1^ according to the vendor’s recommendation (6~9 m·s^−1^). Drop spacing (DS) is a primary parameter that determines printing resolution and uniformity [21]. All silver patterns were printed with DS value set at 20 μm. A silver nanoparticle-based ink (DGP 40LT-15C, ANP Co. Ltd., Sejong, South Korea) was used in this research. This ink consists of silver nanoparticles (30.7 wt. %) and a polar solvent (Triethylene Glycol Dimethyl Ether: C_8_H_18_O_4_). The viscosity and surface tension of the silver ink were 15.4–15.7 mPa·s (measured by Brookfield LVDV-I+, Brookfield, MA, USA) and 40.2 mN·m^−1^ (measured by KRUSS Tensiometer K9, Hamburg, Germany) at room temperature, respectively (data provided by the vendor).

### 2.2. Fabrication of Thin Flexible Parylene ECoG Electrode Array

**Figure 2:**
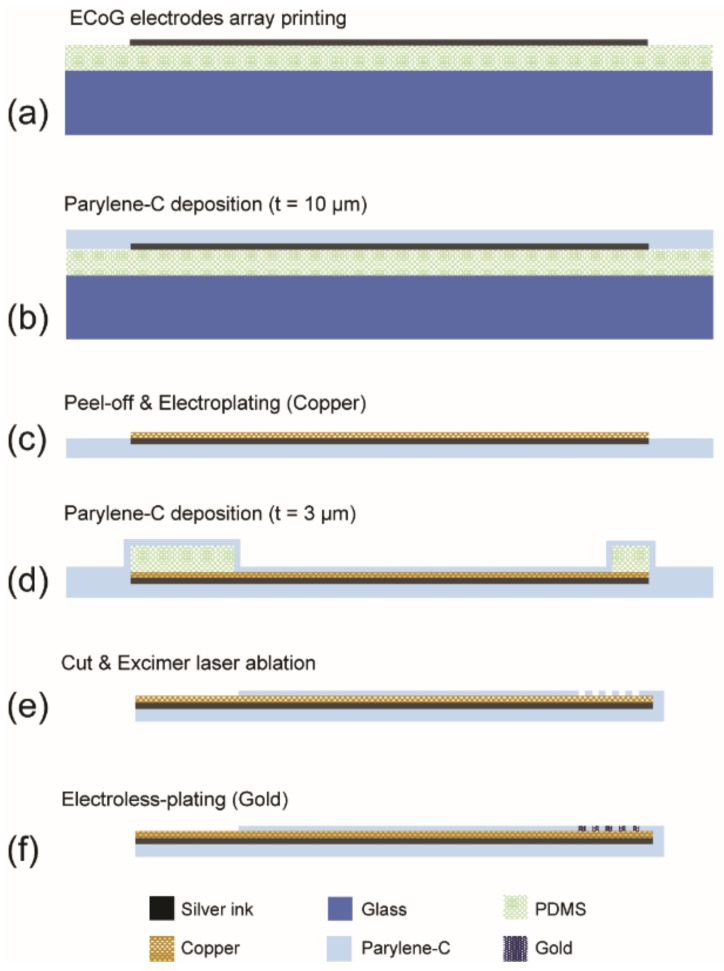
The overall fabrication flow of a thin flexible Parylene ECoG electrode array: (**a**) inkjet printing of silver electrodes on PDMS (spin-coated on a glass substrate); (**b**) Parylene deposition (10 µm); (**c**) peeling off the Parylene layer (silver electrodes are transferred from PDMS to Parylene) followed by the sintering and electroplating of Cu; (**d**) Parylene deposition (3 µm)—electrode pads and reference electrodes are not coated with Parylene by placing PDMS blocks; (**e**) excimer laser machining of Parylene over sensing sites; and (**f**) electroless plating of gold on the sensing sites.

The overall fabrication flow of a thin, flexible Parylene ECoG electrode is shown in Figure 2. The inkjet printing of silver micropatterns onto PDMS layers has been reported by our group [21]. The degassed PDMS mixture (base resin: curing agent = 10:1) was spin-coated on soda-lime glasses (50 × 50 mm) at 500 rpm for 60 s. A thin PDMS layer (~200 µm) was obtained after curing in a convection oven at 60 °C for 1 h. The surface of PDMS is hydrophobic and does not provide a good wettability for polar solvent-based silver ink. Air plasma treatment was used to obtain a hydrophilic PDMS surface and ensure the printing quality for continuous patterns. The PDMS layer was treated with air plasma using an auto plasma system (Femto Science, CUTE-1MPR, Hwaseong, South Korea) at 100 W for 1 min [21]. The ECoG microelectrodes array was inkjet printed on the PDMS layers (Figure 2a). Note that the direct printing of silver patterns on the Parylene surface was investigated in our previous study [37] and only produced very irregular lines on the Parylene surface, showing a lack of control regarding the line width. Air plasma treatment was helpful, but the ink spread was too great to obtain narrow lines. Moreover, the electrical resistances of those lines were also too high for them to be used as electrodes. As an alternative method, transferring the printed silver pattern from PDMS onto Parylene film turned out to be successful and reproducible to create continuous and narrow microelectrodes.

In the next step, a 10 µm-thick Parylene-C was deposited onto the PDMS layer via the vapor deposition method (Specialty Coating Systems, PDS 2010, Melbourne, FL, USA) (Figure 2b). The amount of raw dimer and the operation time required depended on the desired coating thickness. For the current application, 4.5 g of Parylene dimer was used for about 10 µm-thick Parylene film deposition. The Parylene-C film was then gently peeled off from the PDMS substrate. During this process, the printed ECoG microelectrodes array was transferred onto the Parylene-C film from the PDMS. The ECoG microelectrodes made of silver nanoparticle ink were then sintered in a vacuum oven at 180 °C for 1 h. To increase the electrical conductivity of the electrodes, we electroplated a thin copper layer on silver patterns using an electrolyte (Grobet USA, Clean earth Copper mirror, Carlstadt, NJ, USA) and DC power supply (GW Instek, SPS-3610, New Taipei City, Taiwan) at 30 mA for 2 min (Figure 2c). A 3 µm-thick Parylene-C was then deposited for insulation. In this step, 1.5 g of the Parylene dimer were used for Parylene deposition (Figure 2d). The PDMS blocks were placed on the electrode pad area and two reference electrodes to prevent the areas from being coated with Parylene.

The thin Parylene-C layer over the sensing sites was removed without damaging the electrodes using laser ablation. An argon fluoride (ArF) excimer laser was used for the selective opening of sensing sites (Figure 2e). The excimer laser system consists of a micromachining station (Rapid X 250 series, Resonetics, Nashua, NH, USA) and a laser unit (COMPex Pro 110F, Coherent, Germany). The ArF gas mixture (0.17% F2, 5.33% Ar, 16.5% He and Ne) was supplied to the laser unit for generating a 193 nm wavelength excimer laser. The amount of voltage was 25.5 kV under constant energy mode, and the fluence of the ArF excimer laser was 101.56 mJ/cm^2^, using a 50% transmission rate attenuator. Thirty-two shots were applied to each sensing site for the removal of Parylene insulation.

Finally, a thin gold layer was selectively deposited on laser-ablated sensing sites using electroless plating to improve the biocompatibility of the electrodes for in vivo studies (Figure 2f). We used a two-part system (Part A: Part B = 14: 1) to produce thin gold films on the electroplated copper layer. The mixed gold solution (Transene Company Inc., Danvers, MA, USA) was heated on a hot plate at 75 °C. Then, the laser-ablated ECoG electrode array was soaked in the gold solution for 45 min for gold deposition on both the sensing sites and reference electrodes.

### 2.3. Electrical Connector and In Vitro Characterization of ECoG Electrode Array

The fabricated ECoG electrode array needs an electrical connector to be interfaced with a monitoring device. For this purpose, we designed an electrical connector for our flexible Parylene ECoG electrodes using 3D CAD software. The connector consists of two parts (upper and bottom), and both were 3D printed using a stereolithography system (DSP III Standard SXGA+, EnvisionTec, Dearborn, MI, USA). A ceramic-filled photopolymer material (RCP130, EnvisionTec, Dearborn, MI, USA) was used in the process. The ECoG electrode array was placed on the lower connector first, and the assembly was completed by covering it with the upper connector. This 3D printed connector allows easy replacement of the ECoG electrode array.

The impedances of each sensing site of the thin flexible ECoG electrodes were measured using electrochemical impedance spectroscopy (EIS), and a three-electrode setup. ECoG electrodes (working electrodes), gold deposited reference electrodes (area: 2 × 2.25 mm^2^), and an Ag/AgCl reference electrode (CH instrument, CHI111, Bee Cave, TX, USA) were placed in Dulbecco’s phosphate-buffered saline (Mediatech, 1x DPBS, Manassas, VA, USA) at room temperature. The impedance value was measured in the frequency range from 10 to 10^5^ Hz, while a sinusoidal modulated voltage with 100 mV RMS was applied through a potentiostat (Biologic, VSP, Knoxville, TN, USA).

### 2.4. In Vivo Testing of ECoG Electrode Array in a Rat

The surgical implantation of the fabricated ECoG electrode array was conducted using a Lewis rat. The surgical procedures were performed under aseptic conditions at the University of Texas Rio Grande Valley (UTRGV) Animal Facility. Before the implantation, the rat was placed into an induction chamber, and isoflurane (5.0%) was used to induce anesthesia, followed by maintenance of anesthesia (1.0–2.0%) in oxygen until unconscious. The surgery spot was shaved and cleaned at the top of the rat’s head using a betadine scrub and isopropyl alcohol. Then, the rat was secured to a surgery table, and its body temperature was controlled with a hot pad. The scalp on top of the head was removed, and the bone surface was disinfected using hydrogen peroxide. UV-curable dental acrylic was also applied to the periphery of the craniotomy site.

The ECoG electrode array was attached to a stereotaxic arm, and the electrode sites were facing downward and contacting the dura surface. Small pieces of saline-soaked Gelfoam were used to cover exposed dura or pia. In addition, a small amount of saline-soaked Gelfoam was placed on the top of the thin-film electrode. UV-curable dental acrylic was applied to create a stable head cap. After the dental acrylic was completely cured, the skin was sutured around the head cap, and the rat was removed from the stereotaxic frame. Antibiotic ointment was applied around the wound. The flexible ECoG microelectrode array was implanted and maintained for two weeks. All surgical procedures were conducted following the Guide for the Care and Use of Laboratory Animals of the Institute of Laboratory Animal Resources, Commission on Life Sciences, National Research Council (National Academy Press, Washington, DC, USA, 1996) and were reviewed and approved by the Institutional Animal Care and Use Committee UTRGV. The fabricated 22 channels (2 references and 20 sensing channels) were implanted on the cortical surface of the rat.

## 3. Results and Discussion

### 3.1. Fabricated Device (Thin Flexible Parylene ECoG Electrode Array)

**Figure 3:**
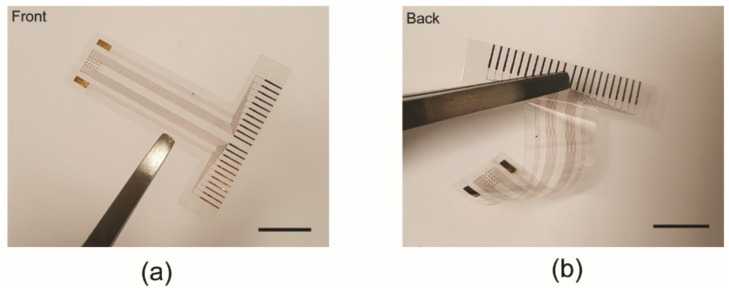
A complete Parylene ECoG electrode array: (**a**) front view of the thin flexible Parylene ECoG electrode array, (**b**) backside of the device showing its flexibility. (Scale bar: 10 mm).

Figure 3 shows a complete Parylene ECoG electrode array. The device is highly flexible and transparent. No wrinkle is generated after bending the device, and the electrodes remain stable and functional. The device weighs only 9 milligrams.

**Figure 4:**
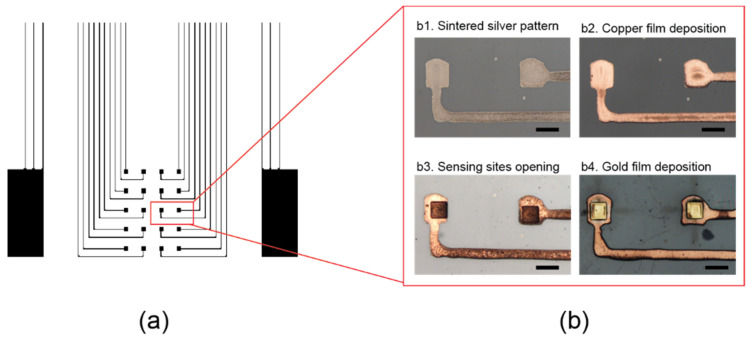
Microscopic images of the sensing sites at different fabrication stages: (**a**) a detailed view of the sensing area of the ECoG electrode array, (**b**) inkjet-printed silver patterns that were transferred to Parylene-C followed by sintering (**1**), thin copper film electroplated onto the silver patterns (**2**), Parylene deposition followed by sensing sites opening by ArF excimer ablation (**3**), and thin gold film deposited on the sensing sites using electroless plating (**4**) (scale bar: 100 µm).

Figure 4 shows the microscopic images of the sensing sites at different fabrication stages. The silver ECoG microelectrodes array printed on PDMS by inkjet printing was transferred to 10 µm-thick Parylene-C film without defects. Different thicknesses of Parylene (1, 5, 10, and 20 µm) were investigated; for all of them, the pattern transfer from PDMS onto the Parylene film turned out to be successful. However, thin Parylene layers could be torn during the peel-off process. In addition, more wrinkles were generated after the peel-off process. Therefore, we chose a 10 µm-thick Parylene layer, which showed a good structural stability and minor wrinkle issues. A thinner Parylene layer (3 µm) was used as the insulation layer. The transferred silver ECoG electrode array was then sintered (Figure 4b(1)) and electroplated with a thin copper layer (Figure 4b(2)). Then, a thin Parylene-C (3 µm) was deposited on the entire surface except for electrode pads and reference electrodes. Next, an ArF excimer laser was used to remove insulation from the sensing sites to create a square-shaped opening (80 × 80 µm), as shown in Figure 4b(3). Finally, to minimize the toxicity of the device for in vivo studies, a thin layer of gold was deposited on top of the copper/silver electrodes at the sensing sites using electroless plating (Figure 4b(4)).

**Figure 5:**
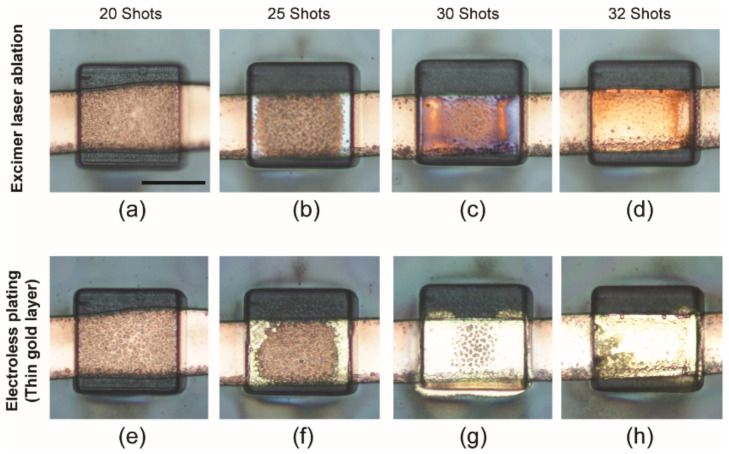
Characterization of laser ablation for sensing site opening and gold deposition: (**a**–**d**) the images of excimer laser-ablated sensing sites at different numbers of laser shots, and (**e**–**h**) the images of the sensing sites after electroless gold deposition (scale bar: 50 µm).

Excimer laser machining offers excellent potential and flexibility for creating high-resolution microstructures that are difficult to make using conventional photolithography-based microfabrication techniques [38,39]. In this study, we used an ArF excimer laser (wavelength = 193 nm, deep ultraviolet light) to ablate a thin Parylene layer for opening the sensing sites because the laser beam is scalable from micrometers to sub-micrometers. For this process, we conducted a characterization study to identify the number of laser shots required to peel off a 3 µm-thick Parylene layer, as shown in Figure 5. The laser was under a constant energy mode with a partial gas replacement function, and an attenuator (transmission rate of 50%) was used. The fluence of the laser was 101.56 mJ·cm^−2^. The number of laser shots was varied from 20 to 32, and we conducted electroless plating of gold with the samples to verify whether the Parylene layer had been completely removed. The laser-ablated samples were soaked in an electroless gold solution at 75 °C for 45 min. We found that Parylene was completely removed with 32 laser shots, as evidenced by the successful gold plating.

### 3.2. Connector Assembly and Impedance Measurement In Vitro

**Figure 6:**
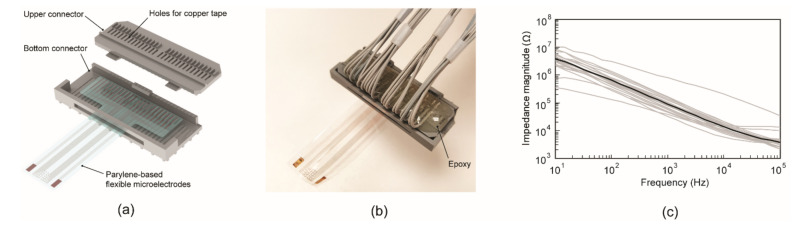
Assembly with an electrical connector and impedance measurement in vitro: (**a**) 3D rendering of the Parylene ECoG electrode array assembled with an electrical connector that consists of top and bottom parts, (**b**) a picture of the fully assembled ECoG signal sensing devices, and (**c**) impedance spectrum for each of the 20 electrodes (gray lines) and the mean spectrum of all electrodes (black line).

The rendering of the electrical connector is shown in Figure 6a. All parts were 3D-printed in less than 3 h. The 3D-printed connector parts required intense light radiation (spectrum range: 300~700 nm) in a chamber for the complete hardening of the material. Then, a narrow, long copper tape was attached to a flat area of each pillar, and the other side was soldered with an electric wire at the top of the upper connector. Twenty-two pieces of copper tape were soldered with wires completely. Finally, the soldered wires were fixed to the connector using epoxy, as shown in Figure 6b. This connector can be used permanently, and the ECoG microelectrode array can be replaced easily without soldering materials and soldering jigs. We tested four-set microelectrode arrays on the connector, and 98.8% of the electrodes remained functional after being assembled with the connector.

The impedance of the assembled ECoG electrodes array was measured by electrochemical impedance spectroscopy. Figure 6c shows the impedance spectral bands of the 20 microelectrodes measured in vitro. The grey curve represents the measurement data of individual electrodes, while the black curve is their average. The average impedance was 90.23 kΩ at 1 kHz. The impedance of the ECoG microelectrodes decreased with the frequency in the range of 10 to 10^5^ Hz. The measured impedance values were several orders lower than the input impedance of the head stage we used for in vivo recording (13 MΩ at 1 kHz, ZD23, Tucker-Davis Technologies, Alachua, FL, USA). This difference in impedance enabled the recording of a high-amplitude signal with a low phase shift [40].

### 3.3. Local Field Potentials Recording by the Implanted Parylene ECoG Electrode Array

**Figure 7:**
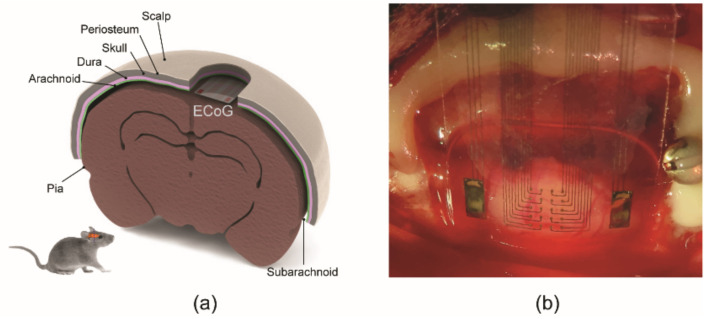
A Parylene ECoG electrode array implanted on the cortical surface of a rat: (**a**) the standard position of ECoG electrodes array for in vivo animal study, and (**b**) the optical image of implanted thin flexible ECoG array for in vivo signal recording.

The fabricated Parylene ECoG electrode array was successfully implanted on the cortical surface of a rat. The standard position of the ECoG array for the in vivo animal study is shown in Figure 7a. Figure 7b shows the signal sensing sites on the cortical surface after implanting the ECoG array.

For signal recording, the soldered wires at the upper connector (Figure 6b) were connected to the TDT Neurophysiology data acquisition system (Tucker-Davis Technologies, Alachua, FL, USA) through the high impedance headstage. A total of 22 channels (2 references and 20 sensing channels) were used for recording. The implanted flexible ECoG electrode array was maintained for two weeks. It was confirmed that the thin flexible ECoG device could successfully record the ECoG signal via the data analysis software (Synapse software, Tucker-Davis Technologies, Alachua, FL, USA).

**Figure 8:**
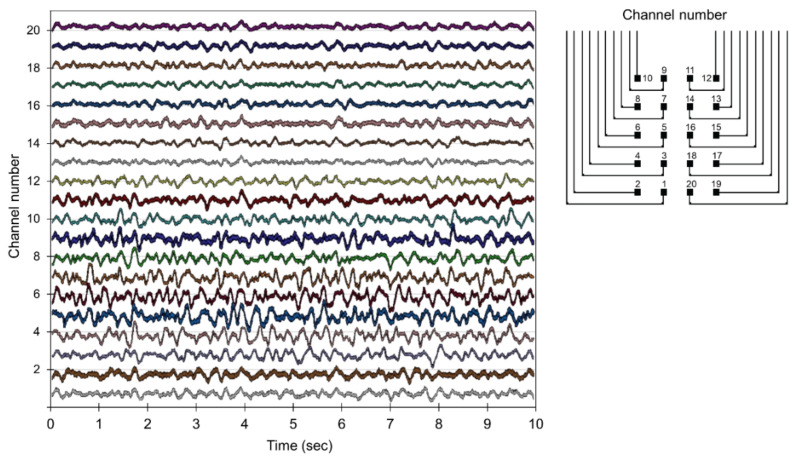
Recorded local field potential (LFP) activity from the rat brain using the flexible ECoG microelectrode array. This representative plot shows 20-channel waveforms together in a 10 s frame of the *x*-axis.

During the experiment, the rat was awake and freely moving in a cage. The local field potential (LFP) activities were successfully detected from 20 microelectrodes distributed over the rat’s brain, as shown in Figure 8. The LFP signals show normal brain activities from awake animals with the peak-to-peak amplitude ranging from 70~160 µV.

## 4. Conclusions

We developed a thin Parylene-based flexible 22-channel ECoG microelectrode array and demonstrated its performances in vitro and in vivo. The narrow (<40 µm) and thin (<500 nm) microelectrode pattern was first inkjet-printed on PDMS, then the pattern was transferred onto Parylene film via vapor deposition and release. Excimer laser ablation and electro-/electroless plating techniques were also employed to create complete ECoG devices. This unique fabrication technology makes it possible to develop flexible Parylene microelectrode arrays without using conventional photolithography-based microfabrication techniques. A custom-designed, 3D-printed connector was also built and assembled with the Parylene-based flexible ECoG microelectrodes array without soldering. To demonstrate the function of our device, we measured the impedance of the assembled ECoG electrode array using electrochemical impedance spectroscopy. The impedance values were consistent among the microelectrodes. In addition, we implanted the printed ECoG sensor in a rat and successfully recorded brain signals in vivo. We believe that Parylene-based flexible microelectrode patterns and the novel fabrication techniques described here can find a myriad of applications in the fields of neuroscience and brain–machine interfaces.

## Data Availability

Not applicable.
